# Imaging spectrum of abnormal subcutaneous and visceral fat distribution

**DOI:** 10.1186/s13244-019-0833-4

**Published:** 2020-02-13

**Authors:** Asako Yamamoto, Yoshinao Kikuchi, Toru Kusakabe, Hideyuki Takano, Keita Sakurai, Shigeru Furui, Hiroshi Oba

**Affiliations:** 1 Department of Radiology, Teikyo University School of Medicine, 2-11-1 Kaga Itabashi-ku, Tokyo, 173-8606 Japan; 2 Department of Pathology, Teikyo University School of Medicine, 2-11-1 Kaga Itabashi-ku, Tokyo, 173-8606 Japan; 3 Department of Endocrinology, Metabolism, and Hypertension Research, Clinical Research Institute, National Hospital Organization Kyoto Medical Center, 1-1 Fukakusa Mukaihata-cho, Fushimi-ku, Kyoto, 612-8555 Japan; 4 Department of Radiology, Chiba Cancer Center, 666-2 Nitonacho, Chuo-ku, Chiba-shi, Chiba, 260-8717 Japan

**Keywords:** Fat, Lipodystrophy, Lipohypertrophy, Lipoatrophy, Bone marrow

## Abstract

Adipose tissue plays multiple and complex roles not only in mechanical cushioning and energy storage but also as an important secretory organ that regulates energy balance and homeostasis multilaterally. Fat tissue is categorized into subcutaneous fat tissue (SCAT) or visceral fat tissue (VSA) depending on its distribution, with the two having different metabolic functions. Near-total lack of fat in congenital/acquired generalized lipodystrophy, cachexia, or any other severe malnutrition condition induces severe multi-organ dysfunction due to lack of production of leptin and other adipokines. Increased visceral fat tissue secondary to obesity, hypercortisolism, or multiple symmetric lipomatosis raises the risk of insulin resistance, cardiac complications, and airway or spinal canal stenosis, although the fat distribution pattern differs in each condition. Partial abnormal fat distribution conditions such as HIV/HAART therapy-associated lipodystrophy, familial partial lipodystrophies, and acquired partial lipodystrophy frequently show a mixture of lipoatrophy and lipohypertrophy with metabolic dysfunction. Characteristic imaging features in conditions with local abnormal fat distribution can provide information about a patient’s co-existent/unrecognized disease(s), past medical history, or lifestyle. Knowledge of characteristic abnormal fat distribution patterns can contribute to proper and timely therapeutic decision-making and patient education.

## Key points

- Adipose tissue plays multiple and complex roles not only in mechanical cushioning and energy storage, but also as a secretory organ that regulates homeostasis.
- It is essential to understand the anatomy and function of fat tissue to facilitate recognition of abnormal conditions and associated disorders.
- Generalized fat loss induces severe multi-organ dysfunction due to lack of production of leptin or other adipokines.
- Generalized or partial excessive fat accumulation can raise the risk of cardiac/respiratory/neurological disorders and insulin-resistance.
- Partial or localized conditions show characteristic distribution patterns on images, possibly shedding light on the patient’s co-existent/hidden disease, past medical history, or lifestyle.

## Background

Until the middle of the twentieth century, adipose tissue was of little interest to scientists, because its sole function was considered to be tissue support [1]. It has now become clear, however, that adipose tissue plays multiple and complex roles not only in mechanical cushioning and energy storage but also as a secretory organ that helps to regulate energy balance, homeostasis, appetite, inflammation, insulin sensitivity, and lipid metabolism [2]. Fat tissue is categorized as subcutaneous fat tissue (SCAT) or visceral fat tissue (VSA) depending on its distribution, and white adipose tissue and brown adipose tissue on its function.

For abnormal fat distribution, terms such as lipodystrophy, lipohypertrophy, or lipoatrophy are used. Lipodystrophy is an umbrella term used to describe a diverse group of metabolic disorders characterized by either complete or partial loss of fat (lipoatrophy), which may occur in conjunction with pathological accumulation of fat in other distinct regions of the body. Conditions that can cause lipodystrophy are classified as genetic or acquired in etiology, but progressing genetic research may change our understanding of their etiology from acquired to genetic or genetic phenotyping in the near future.

There is considerable heterogeneity related to the pattern and extent of fat loss among conditions showing abnormal fat distribution. In this paper, we categorized these conditions into generalized and non-generalized (partial and localized) distribution patterns: those showing abnormal adipose distribution affecting nearly the entire body (generalized), those affecting multiple limbs (partial), and those showing small, discrete areas of involvement (localized). Conditions of generalized abnormal fat deposition including congenital and acquired generalized lipodystrophy exhibit excessive or inadequate fat accumulation not only in SCAT and VAT, but also in the bone marrow. These conditions derive life-threatening metabolic dysfunction. Partial conditions represented by HIV-1/highly active antiretroviral therapy (HAART)-associated lipodystrophy syndrome frequently show an uneven mixture of lipohypertrophy and lipoatrophy. The severity of this complication also varies, and it may be associated with conditions such as insulin-resistance or airway/spinal canal stenosis. The localized pattern, such as that induced by trauma or mechanical stress-related conditions, frequently exhibits characteristic imaging features, which may provide information about a patient’s co-existent disease(s) and lifestyle.

This paper focuses on the abnormal distribution of subcutaneous and visceral adipose tissue, divided into generalized and non-generalized patterns (Table 1). Syndromes associated with vascular malformations can also present as lipohypertrophy but are not discussed here.

**Table 1:** Conditions showing abnormal subcutaneous and visceral fat distribution

	Subcutaneous	Visceral	BM fat loss
Generalized			
** Congenital generalized lipodystrophy**	↓	↓	+
Acquired generalized lipodystrophy	↓	→~↓	±
** Cachexia**	↓	↓	+
** Malnutrition**	↓	↓	+
** Hypercortisolism**	↓	↑	-
** Obesity**	↑	↑	-
Non-generalized			
** Multiple symmetric lipomatosis**	↑	→~↑	
** HIV/HAART therapy-associated**	↑~↓	→~↑	
** Drug injection-associated**	↑(~ ↓)	→	
** Trauma and stress-associated**	↑~↓	→	
Familial partial lipodystrophies	↑~↓	→~↑	
** Autoimmune disease-associated**	↓	→	
Acquired partial lipodystrophy	↓	→~↑	

Bold conditions are shown in figures

*BM* bone marrow

## Normal adipose tissue anatomy, types, and physiology

### Anatomy

Mature adipocytes contain only one-third of adipose tissue. Nerves and vessels, fibroblasts and adipocyte precursor cells contain the remaining two-thirds of adipose tissue [3]. Mature adipocytes are divided into two cytotypes, white and brown, recognized by their colors and functions. White adipocytes are unilocular large spherical droplets [4]. On the other hand, brown adipocytes are multilocular with abundant mitochondria packed with cristae within the cytoplasm.

### White adipose tissue (WAT) and brown adipose tissue (BAT)

It is well known that mammals have two types of adipose tissue: white adipose tissue (WAT), mainly composed of white adipocytes, and brown adipose tissue (BAT), mainly composed of brown adipocytes [2]. They are mixed to various degrees depending on factors such as the individual’s age and circumstances (e.g., starvation and cold exposure). White adipose tissue (WAT) comprises most of adult fat. Although WAT and BAT both receive a vascular and nerve supply, BAT contains a richer vascular tree, dense with numerous capillaries [2].

The physiology of WAT can be summarized into three overlapping categories: lipid metabolism, glucose metabolism, and endocrine function [3]. Leptin, one of the most thoroughly investigated adipokines, is referred to in the next section in congenital generalized lipodystrophy.

In neonates, BAT can be found in several areas including the interscapular region, axillae, and muscles in the neck [5–7]. In adults, no discrete collection of BAT is observed. The function of BAT in adult humans has been investigated but not determined to date.

### Subcutaneous and visceral fat tissue

There are two major anatomic distributions with unique anatomic, metabolic, or endocrine properties: visceral (VAT) and subcutaneous adipose tissue (SCAT). VAT is divided into intraperitoneal and retroperitoneal distribution. SCAT occupies about 80% of all body fat in healthy adults [8]. SCAT is further divided into superficial subcutaneous and deep subcutaneous adipose tissue [9].

The metabolic function of VAT and SCAT is substantially different. For example, visceral adipocytes are more metabolically active and have a greater lipolytic activity compared with SCAT. Adipocytes from VAT are more insulin-resistant than adipocytes from SCAT [10, 11]. SCAT is the major source of leptin production [8]. Superfluous energy accumulates in adipocytes at SCAT, which acts as a metabolic sink. Only when the capacity of SCAT is exceeded or impaired does VAT accumulation occurs.

## Generalized abnormal fat distribution

### Congenital generalized lipodystrophy (Berardinelli-Seip syndrome) (CGL)

Congenital (genetic) lipodystrophy has been reported in about 1000 patients with various types related to the pattern of fat loss and genetic molecular defect [12, 13]. Congenital generalized lipodystrophy is an autosomal recessive trait, with an incidence estimated at less than one in 12 million [13]. They are recognized at birth by a near-total lack of body fat and increased muscular appearance. Markedly low serum levels of leptin and adiponectin secreted from adipose tissue drive insulin resistance and its complications such as diabetes mellitus, dyslipidemia, hepatic steatosis, acanthosis nigricans, polycystic ovarian disease, and hypertension [12].

**Fig. 1:**
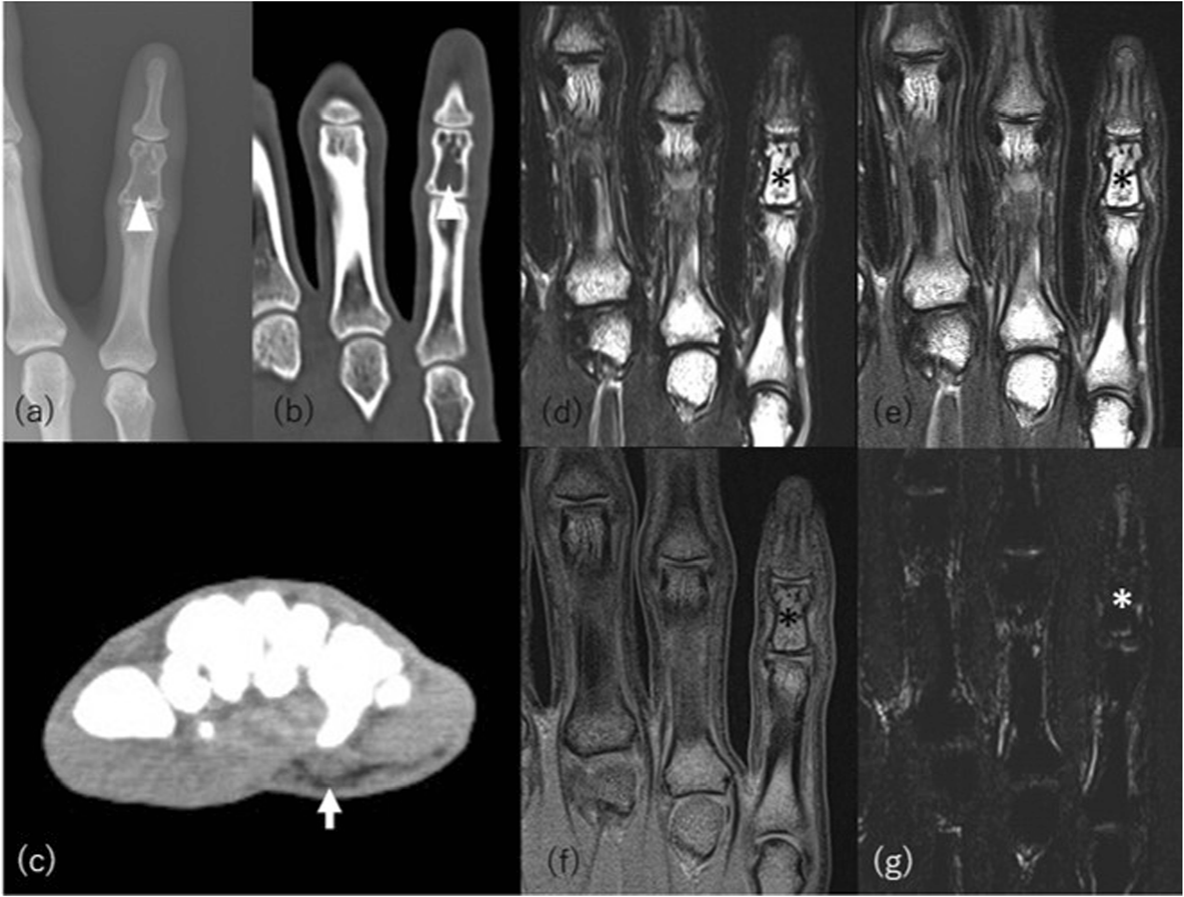
A 23-year-old woman with congenital generalized lipodystrophy and fifth finger pain. Lytic lesion (arrowheads) was evident on (**a**) radiograph and (**b**) CT in the fifth middle phalange. **c** CT showed almost complete loss of fat tissue other than the palmar fat pad (arrow). MRI showed complete lack of bone marrow adipose tissue. Coronal (**d**) T2-weighted and (**e**) STIR images show almost the same signal. **f** T1-weighted image shows no adipose high signal intensity. An asterisk denotes the referred lytic lesion. **g** DIXON-based fat image shows almost complete loss of fat high signal in the bone marrow and soft tissue

**Fig. 2:**
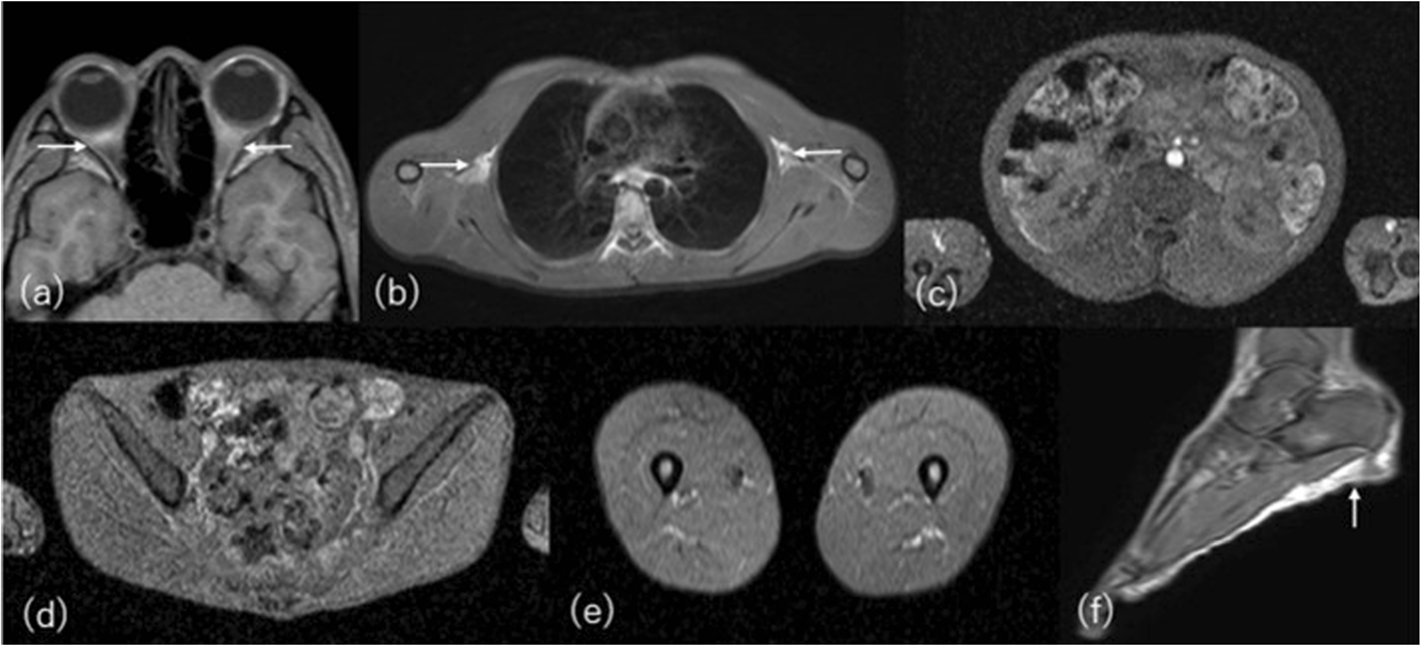
Whole body MRI taken from the same CGL patient 10 years earlier. T1-weighted axial images at the level of (**a**) orbita, (**b**) axilla, (**c**) renal sinus, (**d**) pelvis, (**e**) thigh, and (**f**) sagittal image of foot show nearly complete absence of adipose tissue, which is however slightly preserved in the orbits, axillae, and soles (arrows)

CT and MRI demonstrate the nearly complete absence of adipose tissue well, whereas adipose tissue may be preserved in the orbits, mouth, tongue, palms and soles, scalp, perineum, and periarticular regions [14] (Figs. 1 and 2). Lytic lesions may be formed in the long bones or appendicular bones after puberty [15]. Disappearance of adipose tissue from bone marrow should be anticipated on MRI, which may make any bone lesion difficult to detect. The imaging differential diagnoses are Werner syndrome, anorexia nervosa, cachexia, and other severe wasting conditions. The finding of normal or hypertrophic muscles and past history including medication history are key points for making the diagnosis. Acquired generalized lipodystrophy may show almost the same clinical and imaging findings although the onset is usually during childhood and the distribution and extent of fat loss are variable. Genes for congenital lipodystrophies including CGL continue to be reported [12]. Although there are many types of inherited partial lipodystrophy, most of them are extremely rare. Familial partial dystrophy and acquired partial lipodystrophy are mentioned briefly in the chapter of uneven lipodystrophy.

### Acquired generalized lipodystrophy (AGL)/Lawrence syndrome

AGL is a very rare acquired form of generalized lipodystrophy that develops in a previously healthy child or adult. Loss of SCAT occurs usually during childhood in patients with AGL showing a variable pattern and extent [12]. Although most patients show generalized loss of fat, some also show spared areas such as VAT and bone marrow fat. Severe hepatic steatosis, fibrosis, and diabetes mellitus are the major complication in AGL. The pathogenesis of fat loss in patients with AGL remains unknown, but previous infection can be linked to this syndrome because histologic analysis of subcutaneous tissue reveals panniculitis [16]. Antibodies against adipocyte-membrane antigens have been detected in a few cases. AGL may coexist with autoimmune diseases such as Hashimoto’s thyroiditis, rheumatoid arthritis, hemolytic anemia, and chronic active hepatitis [2].

### Cachexia

**Fig. 3:**
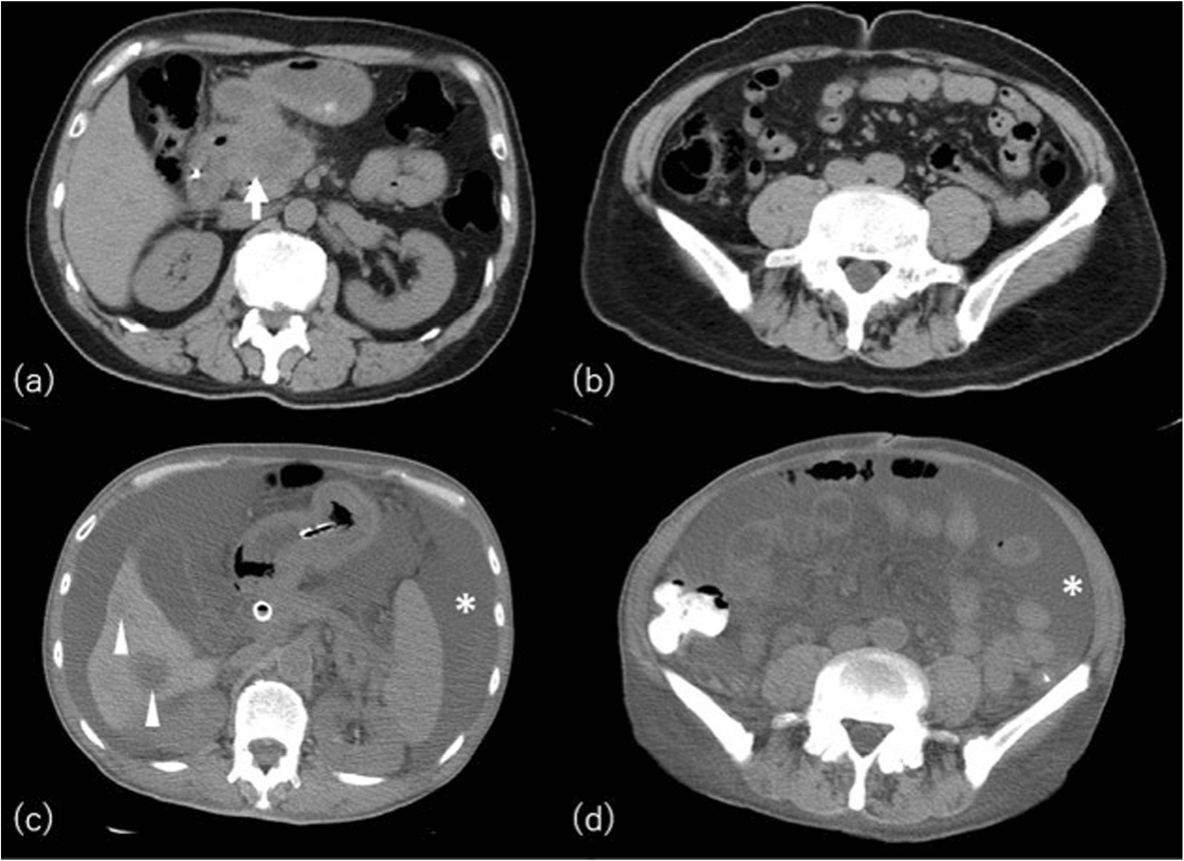
A 68-year-old man with pancreatic cancer. Initial CT in our hospital (**a**, **b**) shows massive low-density mass in the head of the pancreas (arrow). CT performed 14 months later (2 months before death) (**c**, **d**) shows depletion and increased attenuation of subcutaneous and visceral adipose tissue. Hepatic metastases (arrowheads) and copious ascites (asterisks) are also observed

Cachexia is clinically defined as > 5% weight loss over 3 months or > 10% within the previous 6 months in patients with malignancy [17]. Fat loss is a poor prognostic factor in advanced cancer regardless of a patient’s body weight [18]. Adipose tissue metabolism and whole-body fat mass are regulated through two major pathways: lipolysis (fat breakdown) and lipogenesis (fat synthesis). Adipose atrophy in cancer patients is attributed to increased lipolysis and fat oxidation, decreased lipogenesis, impaired lipid deposition and adipogenesis, and browning of white adipose tissue [17]. Cancer patients exhibit smaller adipocytes compared with non-cancer ones, but the total fat cell number is not altered [19]. Fat loss occurs more rapidly and precociously than the reduction of lean mass in cachexia, with extension in rush especially in the immediate period preceding death [20]. CT shows not only fat depletion but also increased density of visceral and subcutaneous fat that may reflect depletion of fat cell size with fibrotic, inflammatory, or edematous change (Fig. 3).

### Eating disorder

Eating disorders are characterized by a persistent disturbance of eating that impairs health or psychosocial functioning with the highest mortality of any psychiatric disorder [21]. Anorexia nervosa and bulimia nervosa are serious life-threatening eating disorders. Anorexia nervosa is characterized by self-starvation and excessive weight loss. Bulimia nervosa is characterized by a cycle of binging and compensatory behaviors such as self-induced vomiting designed to undo or compensate for the effects of binge eating.

**Fig. 4:**
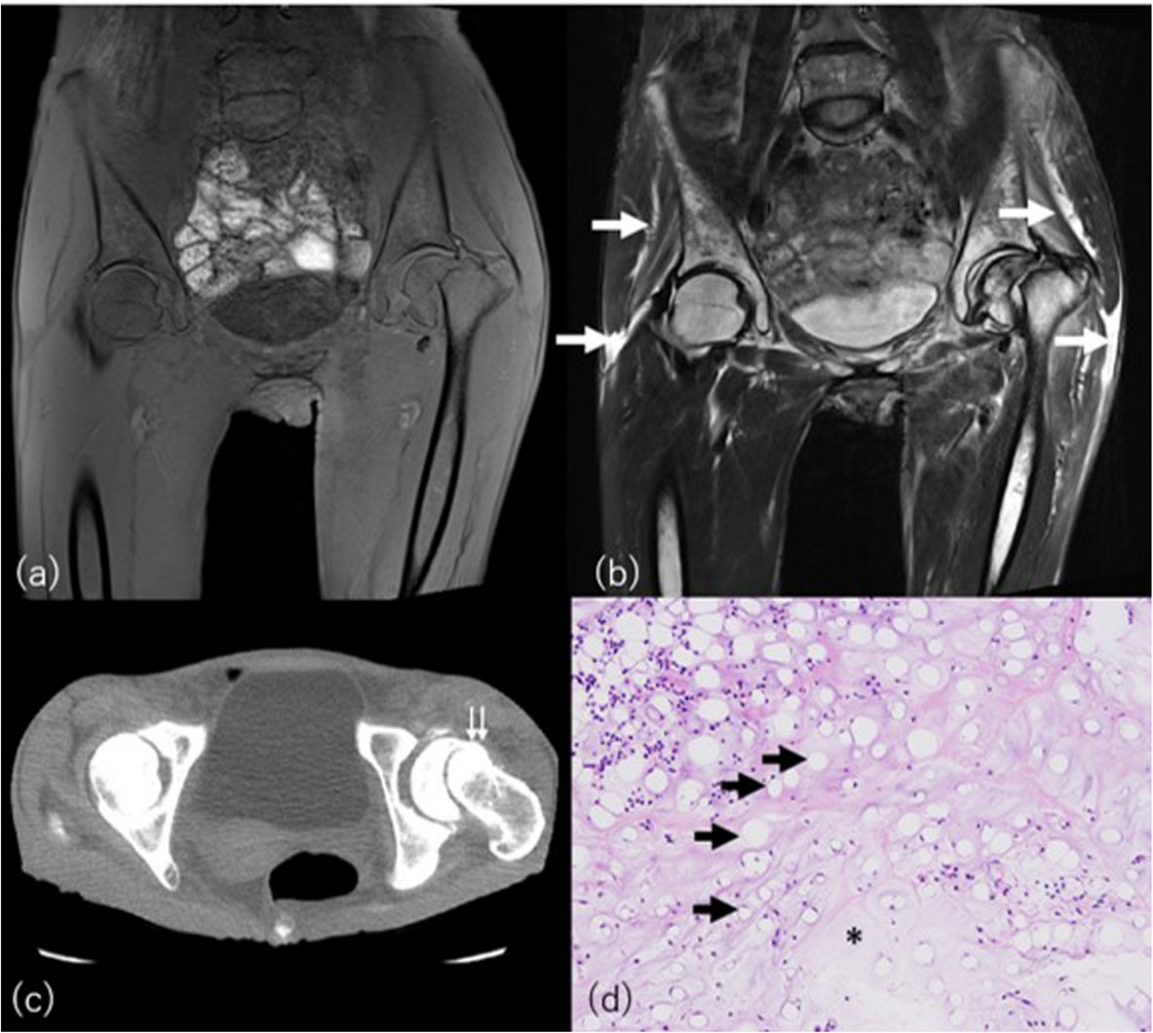
A 48-year-old woman with serous atrophy of bone marrow from bulimia nervosa. **a** Coronal T1-weighted image demonstrates abnormal diffuse hypointense marrow signal with fracture of left femoral neck. **b** Coronal STIR demonstrates abnormal hyperintense signal in the bone marrow and surrounding subcutaneous or intermuscular tissue (arrows). **c** CT at the level of the femoral fracture (double arrow) shows depleted high attenuated fat tissue. **d** Histopathologically shrinkage of fat cells (black arrows), decrease of hematopoietic cells, and deposition of abundant amorphous extracellular gelatinous substances (asterisk) were observed (hematoxylin-eosin stain, × 100)

Patients with severe anorexia or bulimia nervosa can show extremely depleted body fat mass with increased attenuation on CT [22]. Visceral adipose tissue attenuation can be a biomarker of current and prior disease status [22]. Not only subcutaneous and visceral fat, but also diminishment of the normal hyperintense T1-weighted signal is observed from bone marrow (Fig. 4). The signal alteration of bone marrow is named as serous atrophy of bone marrow radiologically or gelatinous bone marrow pathologically. A variety of conditions such as chronic infection, malabsorption, chronic heart and kidney failure, and alcoholism have been identified as causes of serous atrophy of bone marrow [23]. Skeletal growth retardation, in particular in young male patients, may also be evident on imaging studies. Osteoporosis occurs in up to 50% of patients with anorexia with seven times higher the risk of insufficient fracture in patients who have had anorexia for longer than 5 years than in healthy women of the same age [24].

**Fig. 5:**
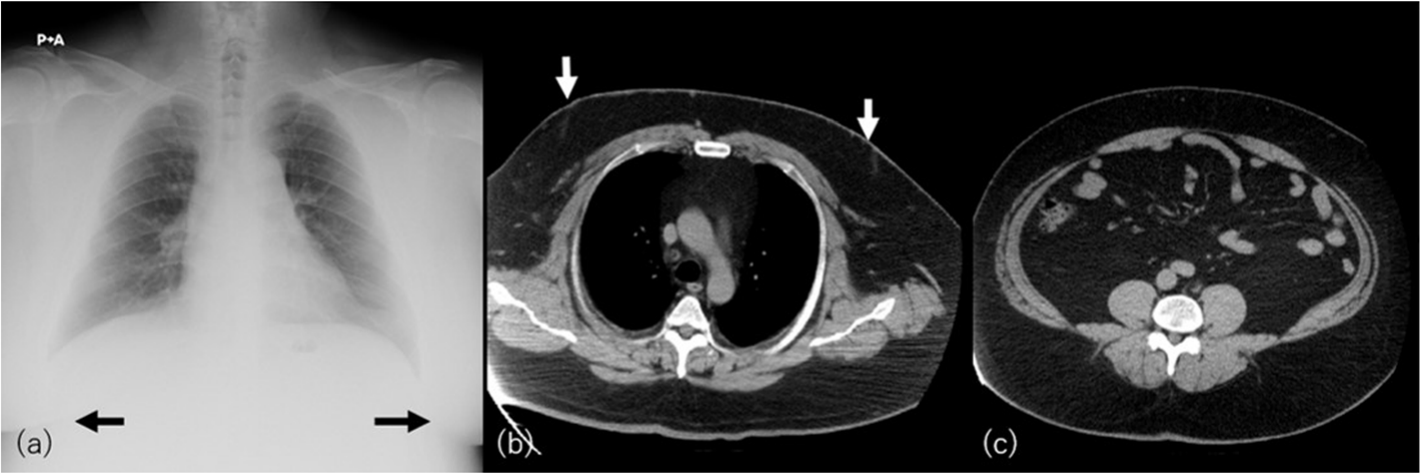
A 37-year-old man with binge eating disorder and overeating habitus (BMI = 53.2). **a** Chest radiograph shows bilateral pseudogynecomastia (black arrows). **b** CT of the chest and (**c**) abdomen shows increase of subcutaneous and visceral adipose tissue. Note the absence of extensive growth of mammary gland but pure fat deposition on the breast (white arrows)

Although obesity (BMI > 30) is not a single but multifactorial condition, binge eating disorder or overeating is one of the most important backgrounds. The increase of fat amount is characterized by increased adipocyte size. Subcutaneous and visceral fat amount is strongly associated with insulin resistance [25]. In obese patients, mass-like subcutaneous fat accumulation can be observed: pseudogynecomastia on the anterior chest wall and buffalo’s hump on the posterior neck. In pseudogynecomastia, the underlying mass is not breast tissue but pure fat (Fig. 5). Buffalo hump can also occur in the course of HIV/HAART therapy, Cushing’s syndrome, or long-time steroid use. Obesity and such conditions may overlap.

## Non-generalized (partial/localized) abnormal fat distribution

A mixture of fat accumulation and lipoatrophy can occur in patients with this category.

### HIV-1/highly active antiretroviral therapy (HAART)-associated lipodystrophy syndrome (HALS)

**Fig. 6:**
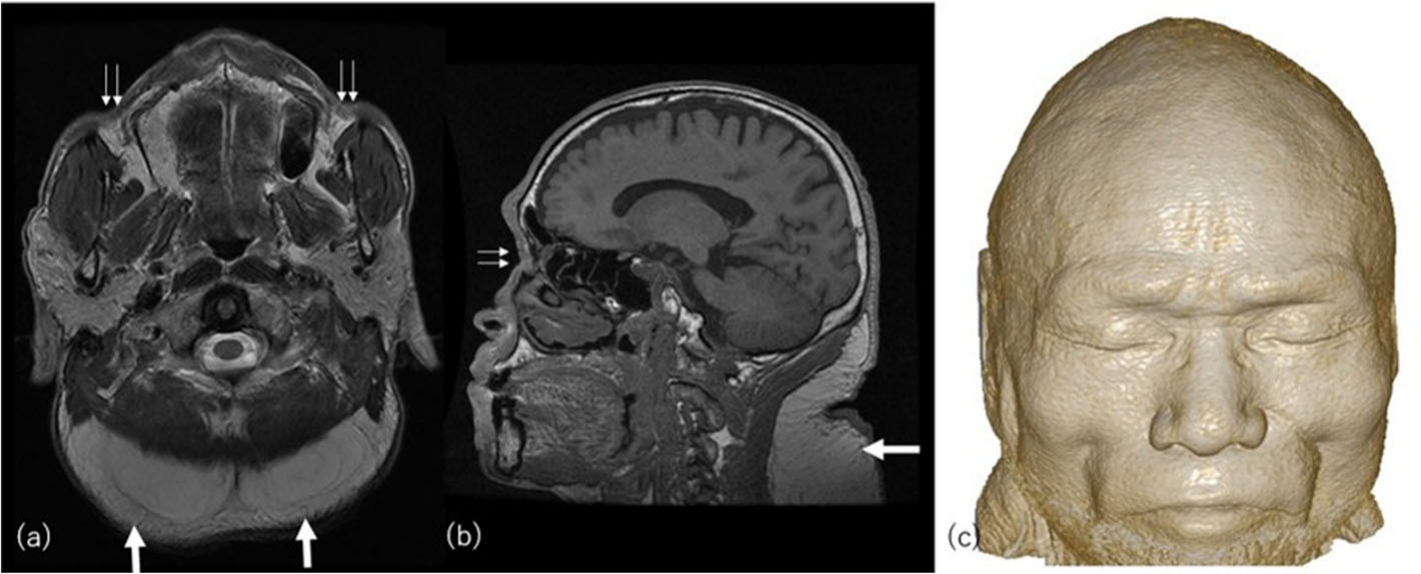
A 47-year-old man with HALS. **a** Axial T2-weighted and (**b**) sagittal T1-weighted images show subcutaneous lipohypertrophy on the posterior neck (arrows). Marked loss of subcutaneous fat including buccal fat pad is shown in the face (arrowheads). **c** Volume-rendered three-dimensional reformation image shows apparent concave cheeks and periorbital hollowing

With the introduction of effective antiretroviral therapy, HALS has become one of its most important long-term adverse effects [26]. HALS includes subcutaneous fat loss (lipoatrophy), fat accumulation (lipohypertrophy), or a combination of both (Fig. 6). These morphologic abnormalities can also be associated with disorders of glucose and lipid metabolism.

Lipoatrophy manifests as a loss of fat mainly in the face, limbs, and buttocks [27]. Facial lipoatrophy is characterized by loss of the buccal and/or temporal fat pads, resulting in concave cheeks, prominent nasolabial folds, and periorbital hollowing [28]. It has been reported that an important fat loss has to exceed 30% to become evident clinically. The main risk factor for lipoatrophy is exposure to thymidine analog nucleoside reverse transcriptase inhibitors (NRTIs), which today, however, are no longer the first-line drugs resorted to [27].

Fat accumulation occurs in the trunk, cervical-dorsal area (“buffalo hump”), breasts, upper chest, or visceral adipose tissue. Dyslipidemia and insulin resistance frequently co-exist in this condition. Risk factors for the development of fat deposition include increasing age, female sex, elevated baseline triglycerides, and higher body fat percentage [29].

### Insulin injection

**Fig. 7:**
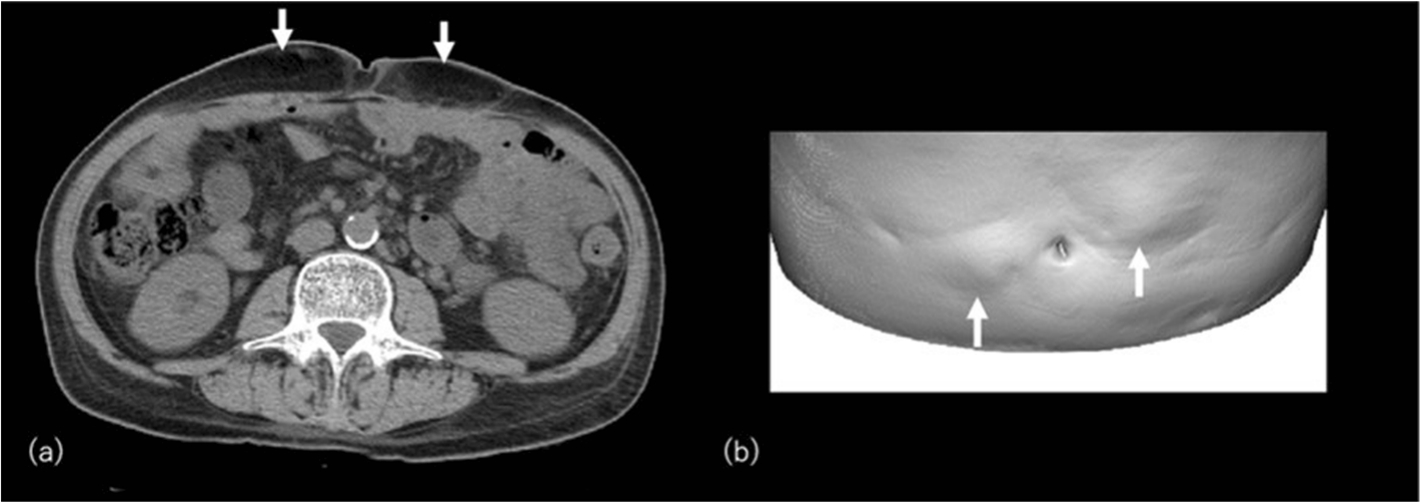
A 66-year-old man with type I diabetes and insulin injection lipohypertrophy. **a** CT image at the level of belly button shows bilateral localized subcutaneous fat deposition on the anterior abdominal wall (arrows). **b** Volume-rendered three-dimensional reformation image show bilateral bulging of abdominal wall reflecting the insulin lipohypertrophy

Abnormal reaction of subcutaneous fat to insulin injection is well known as lipohypertrophy and lipoatrophy. Insulin lipohypertrophy shows a tumor-like swelling of fatty tissue at the injection site due to the lipogenic effect of insulin (Fig. 7). Growth hormone receptor antagonist is also reported to be a cause of lipohypertrophy at the injection site [30]. Lipoatrophy, which is considered to be an immune complex-mediated inflammatory lesion, rarely occurs today since the advent of recombinant human insulin and insulin analogs [31]. Lipohypertrophy remains a frequent complication of insulin therapy, reportedly in 28.7% of those with type 1 diabetes and 3.6% with type 2 diabetes with a greater tendency of developing the lesion in patients using medium or long-acting insulin [32, 33]. Injection into lipohypertrophied sites may contribute to poor glycemic control due to an erratic absorption of the drug.

### Multiple symmetric lipomatosis (Madelung’s disease) (MSL)

**Fig. 8:**
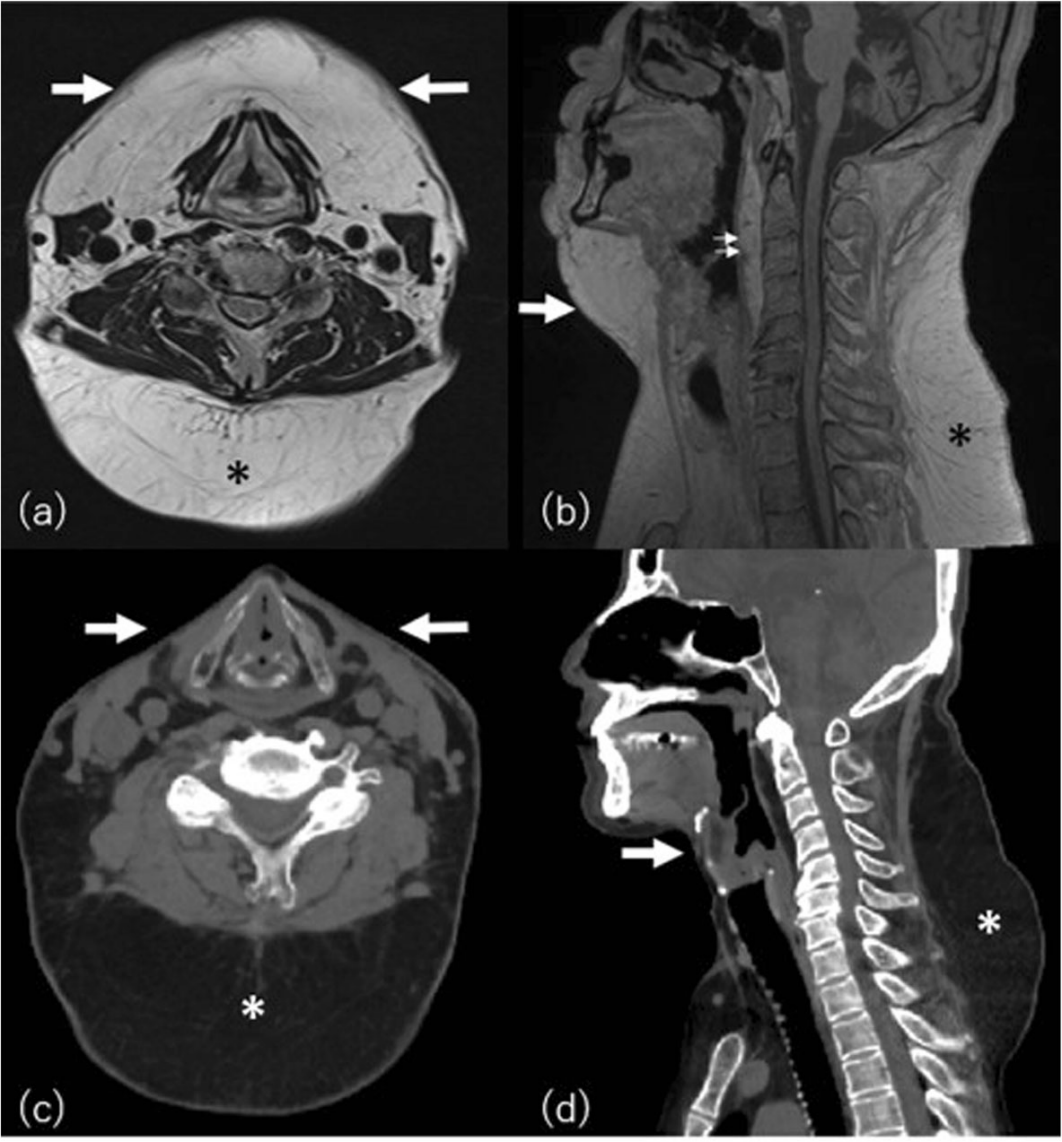
A 45-year-old man with multiple symmetrical lipomatosis with sleep apnea. **a** Axial T2-weighted and (**b**) T1-weighted image show abnormal subcutaneous fat deposition in the anterior (arrows) and posterior neck (asterisks). Note the increase of adipose tissue also observed in the posterior pharyngeal space (small arrows). **c** Post liposuction axial and (**d**) sagittal reconstructed CT 2 years later. Subcutaneous fat volume in the anterior neck is markedly decreased (arrows), whereas in contrast, the fat deposition in the posterior neck has worsened (asterisk)

Madelung’s disease, also known as benign symmetric lipomatosis, is a rare condition that shows a symmetrical accumulation of adipose tissue, primarily around the neck, back, shoulders, and upper trunk (Fig. 8). It usually affects adult males, mostly in the Mediterranean and eastern European population aged from 40 to 50 years [34]. MSL has a strong association with heavy alcohol intake. Alcohol cessation and weight control are recommended although they do not reverse or stop the course of the disease [35]. MSL is also found in non-alcoholics in association with mitochondrial DNA mutations [36]. A defect of adrenergic stimulated lipolysis or mitochondrial disorder of brown fat tissue has been considered as the etiology of this disease in recent studies [37].

Suprascapular and supraclavicular involvement is common and tracheal or esophageal compression due to deep space-occupying lesions is a life-threatening complication [38]. CT is the optimal modality to evaluate deep-seated fat accumulation. Palliative removal of fatty tissue by surgical resection or liposuction and by injection lipolysis is recommended when symptomatic.

### Cushing’s syndrome/hypercortisolism

**Fig. 9:**
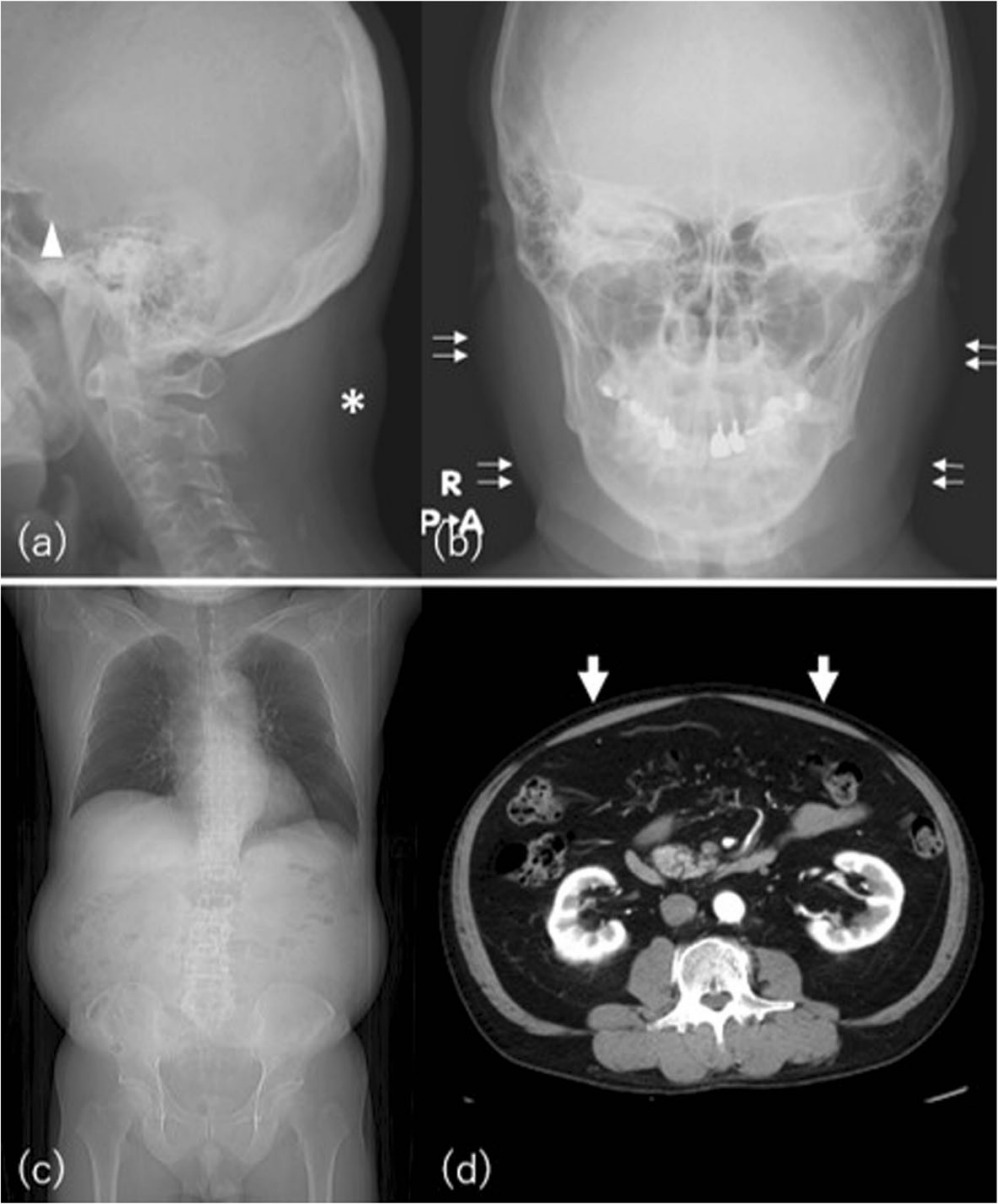
A 52-year-old man with Cushing disease. **a** Lateral skull radiography shows “buffalo hump” on the posterior neck (asterisk). Note the enlarged sella due to pituitary macroadenoma (arrowhead). **b** Frontal skull radiograph shows a rounded shape of the face, “moon face” (double arrows). **c** CT scanogram shows characteristic central obesity. **d** CT image at the level of bilateral renal hilum shows marked visceral fat deposition, in contrast to subcutaneous fat thinning (boxed arrows)

Cushing’s syndrome results directly from chronic exposure to excess glucocorticoid. It includes iatrogenic, ectopic adrenocorticotropic hormone (ACTH) syndrome, Cushing disease (pituitary ACTH-dependent Cushing’s syndrome), and adrenal tumors [39]. The most common feature of patients with Cushing’s syndrome is progressive central obesity (Fig. 9). Adipocytes from VAT are more insulin-resistant compared with SCAT. The amount of visceral fat is a striking factor underlying the enhanced cardiovascular risk seen in this condition and is mediated by insulin resistance [40, 41]. Central obesity can also induce hypertension through increased activity of adipose tissue renin-angiotensin-aldosterone system, sympathetic activation, and other mechanisms connected with insulin resistance.

The extremities are usually spared and may be wasted. Fat can also accumulate in the supraclavicular fossa, spinal canal (spinal epidural lipomatosis), cheeks (moon face), or dorsocervical fat pad (buffalo hump) [39, 42]. Buffalo hump in Cushing’s syndrome is usually consistent with the general degree of obesity. In the muscle, weakness and proximal muscle wasting are induced by the catabolic effects of excess glucocorticoid on the skeletal muscle. Osteoporosis is caused by decreased intestinal and renal calcium absorption and increased bone resorption [43].

### Autoimmune disease

**Fig. 10:**
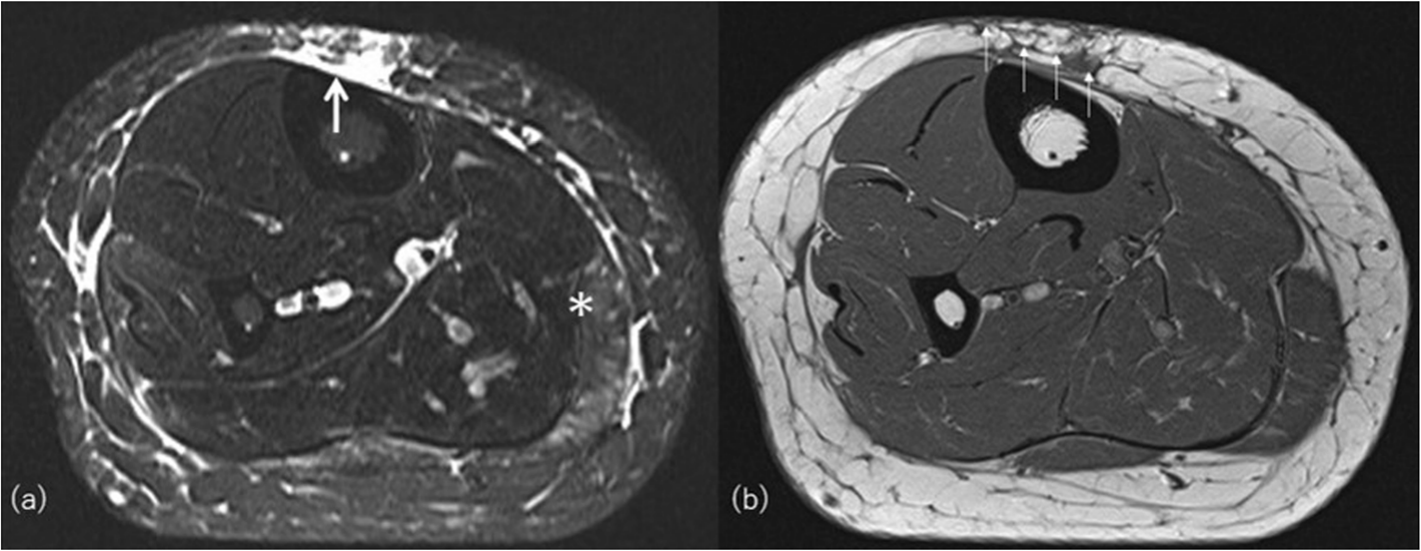
A 48-year-old woman with deep morphea on her right thigh. **a** Axial fat saturated T2-weighted image shows hypersignal intensity in full thickness of subcutaneous fat on the anterior aspect (arrow). Increased signal intensity in gastrocnemius muscle (asterisks), muscle fasciae, and diffuse subcutaneous septal thickening are also demonstrated. **b** T1-weighted image clearly shows thinning of subcutaneous fat with cord-like low signal intensity (small arrows)

Localized scleroderma (morphea) and lupus erythematosus panniculitis (lupus erythematosus profundus (LEP)) are well-known connective tissue disorders involving the subcutaneous compartment resulting in lipoatrophy [44]. They are usually localized but rarely progress to acquired generalized lipoatrophy [12]. Morphea is classically confined to the skin and/or underlying tissues but may extend over muscular fascia, muscle tissue, tendons, joint synovia, and even bone marrow (deep morphea) (Fig. 10). Morphea usually manifests as a single well-circumscribed lesion on the extremities or upper trunk, near the spine with keloid-like hard and shiny skin changes [45]. MRI shows localized lipoatrophy under the depressed thickened skin with or without varying degree of signal change in underlying fascia and musculature involvement. It is usually asymptomatic and not associated with visceral involvement. Generalized morphea is the most severe form of localized morphea. Clinical diagnostic criteria are applied for diagnosis: four or more lesions larger than 3 cm in diameter or involvement of two or more of the seven body areas (the head and neck, the right and left upper extremities, the anterior and posterior trunks) [45].

**Fig. 11:**
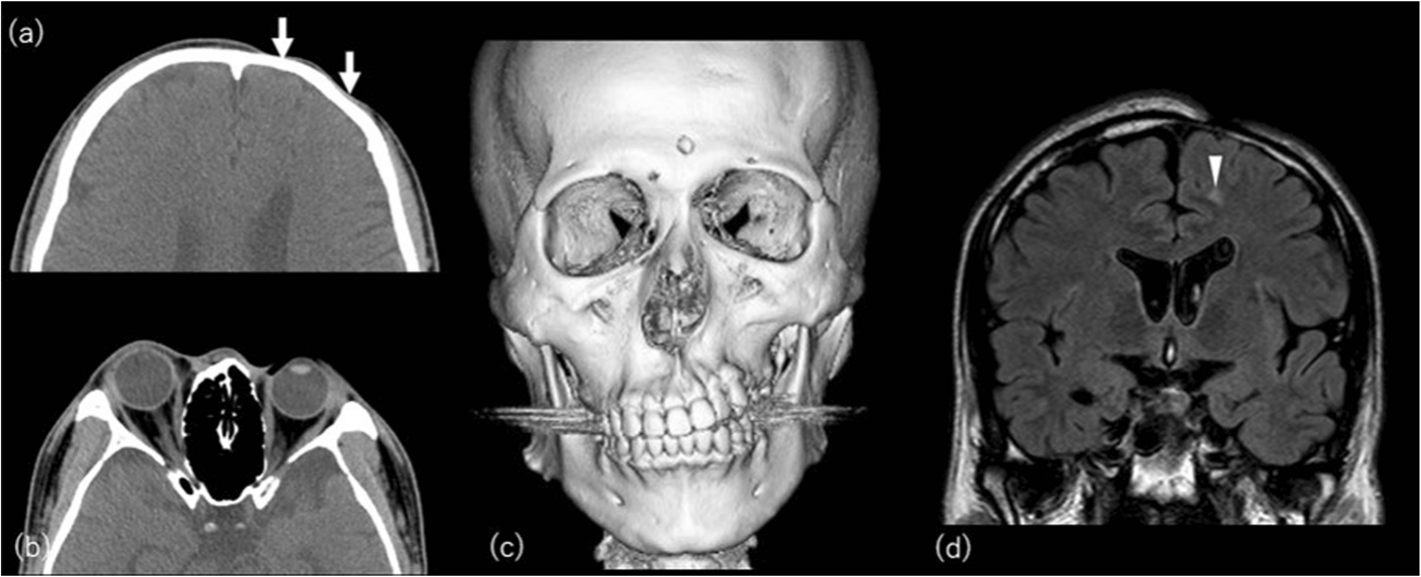
A 29-year-old man with Parry-Romberg syndrome. **a** Axial CT images show asymmetrically decreased subcutaneous fat on the left side (arrows). **b** Hypoplasty of the left orbit is also evident. **c** Volume-rendered three-dimensional reformation image of facial bones highlights bony asymmetry of the face. There is a perceivable asymmetry of the maxillary bone as well. **d** Coronal FLAIR image shows subcortical high signal intensity area (arrowhead)

Linear scleroderma is characterized by one or more linear streaks of cutaneous induration that may involve the dermis, subcutaneous tissue, muscle, and underlying bone. It affects mainly children and is more common in females than males (3:1) and named “en coup de sabre” (LScs) when affecting the hemiface [46]. Parry-Romberg syndrome (PRS), also known as progressive hemifacial atrophy, clinically overlaps with LScs and can even affect the brain [47]. CT and MRI may show cerebral hemiatrophy or high signal of white matter on T2-weighted image ipsilateral to the affected facial side not only atrophy of the skin and underlying bone and soft tissue (Fig. 11) [48].

LEP is an unusual variant of lupus erythematosus (LE), which occurs in 1–3% of patients with cutaneous LE [49]. LEP primarily involves subcutaneous tissues and tends to have a chronic course resulting in broad lipoatrophy. Face and limbs are most commonly involved [50].

### Stress/trauma-induced

#### Post-traumatic lipoatrophy/hypertrophy

**Fig. 12:**
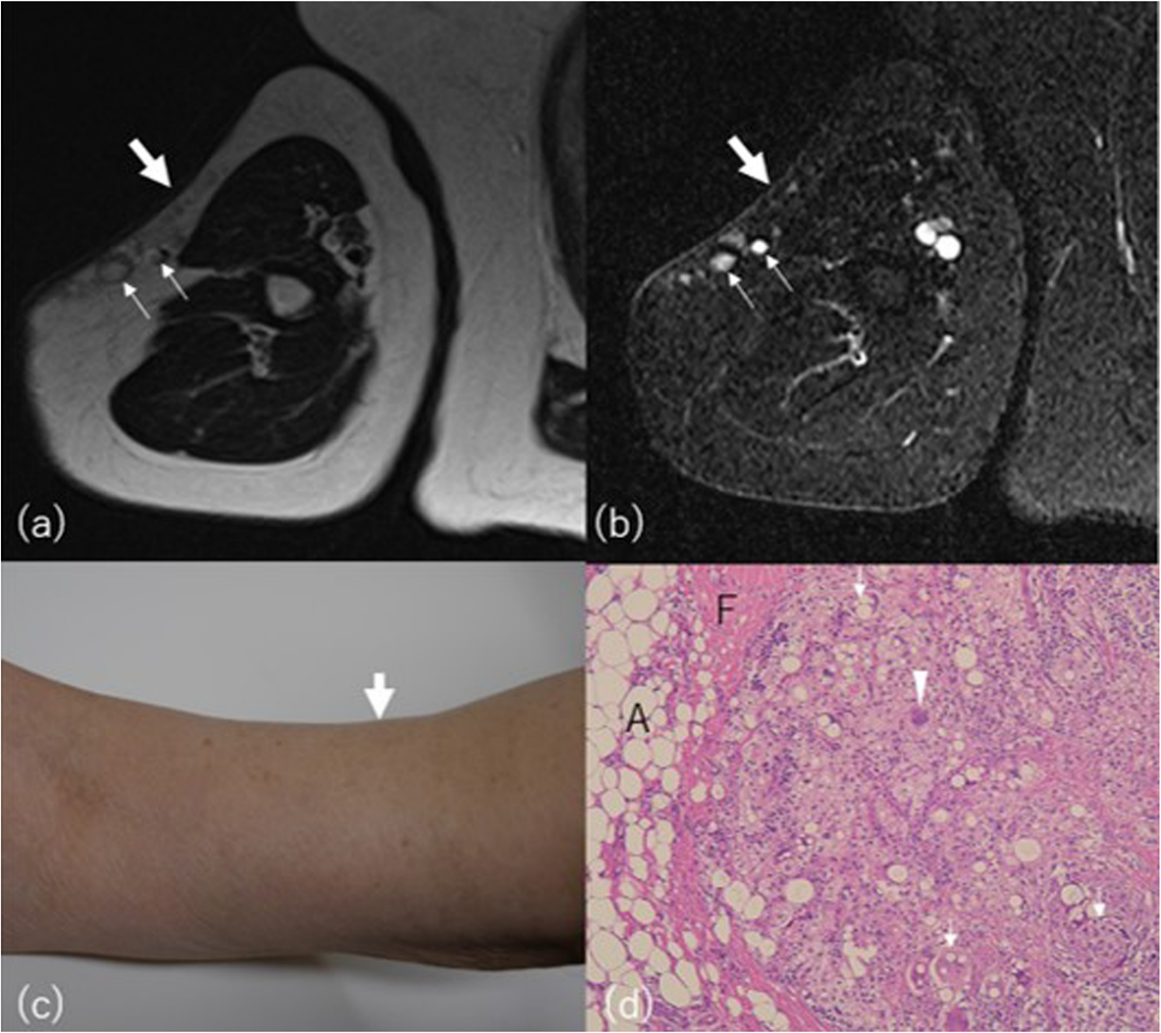
A 59-year-old woman with post blunt trauma lipoatrophy on the right upper arm. **a** Axial T2-weighted and (**b**) STIR images show thinning of subcutaneous fat on the lateral aspects (arrows) with multiple high signal nodules (small arrows) showing small peripheral fat signal areas. **c** Photograph shows thinning of the lateral aspect of the upper arm with small hump (arrow). **d** Histologically fat necrosis with lipogranuloma was proven. Variably sized lipid vacuoles are surrounded by foam cells, foreign body-type (arrows), and Touton giant cells (arrowhead) in the resected lipogranuloma. *A* adipocytes, *F* fibrosis (hematoxylin-eosin stain, × 100)

**Fig. 13:**
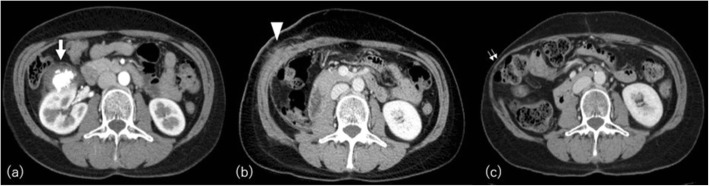
A 59-year-old woman with post-surgery lipoatrophy. **a** CT image before operation shows calcified soft tissue density mass anterior to the right kidney (arrow). Right nephrectomy was done, and the lesion was pathologically diagnosed as dedifferentiated liposarcoma. **b** CT image one month after surgery shows fluffy opacity in the subcutaneous fat around the operated area (arrowhead) with abdominal wall muscle swelling. **c** CT image after 8 years shows local lipoatrophy (double arrow) with muscle atrophy

Posttraumatic subcutaneous lipoatrophy occurs following a fall, blunt injury (Fig. 12), or surgery (Fig. 13), with subsequent fat tissue damage, organized hemorrhage, fat necrosis, and fibrosis [51]. Often, the interval between the injury and initial observation of the deformity is prolonged. It is more prevalent in women and children, usually appearing on the shins, thighs, arms, breasts, and buttocks [52]. The radiologic appearance of subcutaneous posttraumatic lipoatrophy may accompany linear spiculated lesion with globular component on MRI, correlated with lipogranuloma pathologically [53, 54].

**Fig. 14:**
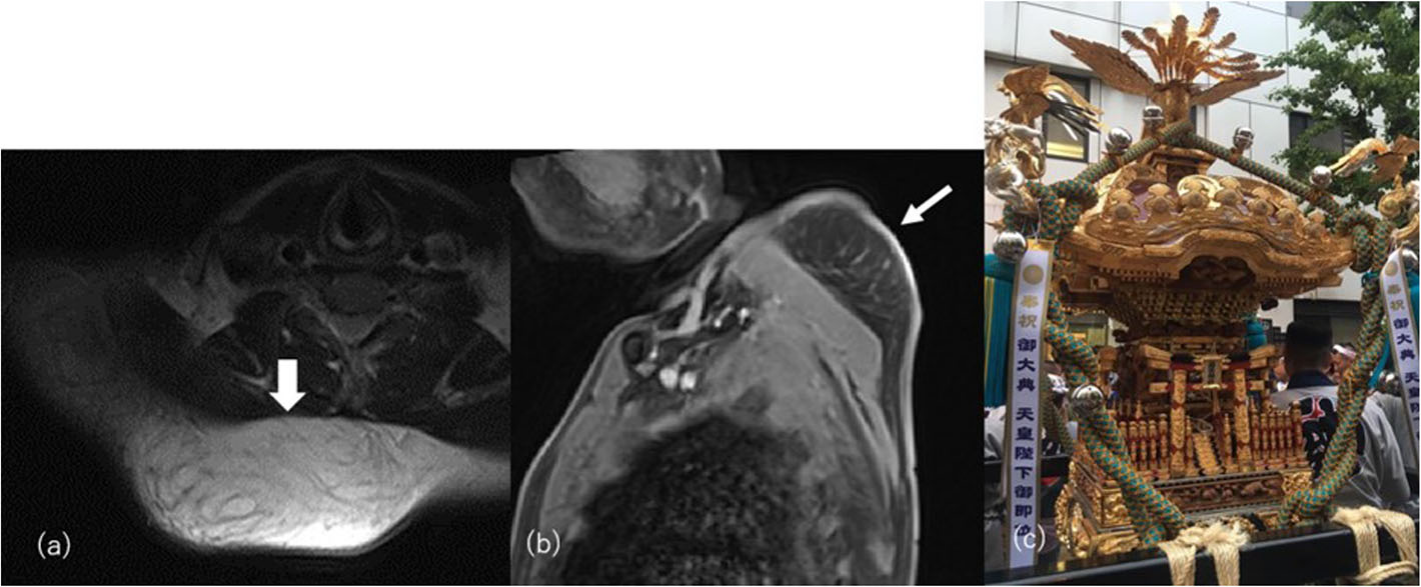
A 60-year-old man with post-traumatic pseudolipoma on the right posterior neck. He had a history of carrying a mikoshi (Japanese portable shrine) daily. **a** Axial T1-weighted and **b** sagittal post-contrast fat-sat T1-weighted images show non-capsulated subcutaneous fat tissue proliferation on the right posterior neck (arrows). **c** Photograph of a mikoshi

Although the pathogenesis of post-traumatic lipohypertrophy, so-called “pseudolipoma,” is not well-proven, blunt trauma has classically been reported as the cause [55]. Repetitive mechanical stress also induces a prominent increase in the volume of subcutaneous adipose tissue. By carrying heavy loads, abnormal local fat accumulation on the shoulder has been reported in festival participants in Japan and Southern Italy, wine porters, brewery workers, and heavy handbag carriers [56]. On CT and MRI, an increase in the volume of non-capsulated adipose tissue is evident (Fig. 14).

#### Semicircular lipoatrophy

**Fig. 15:**
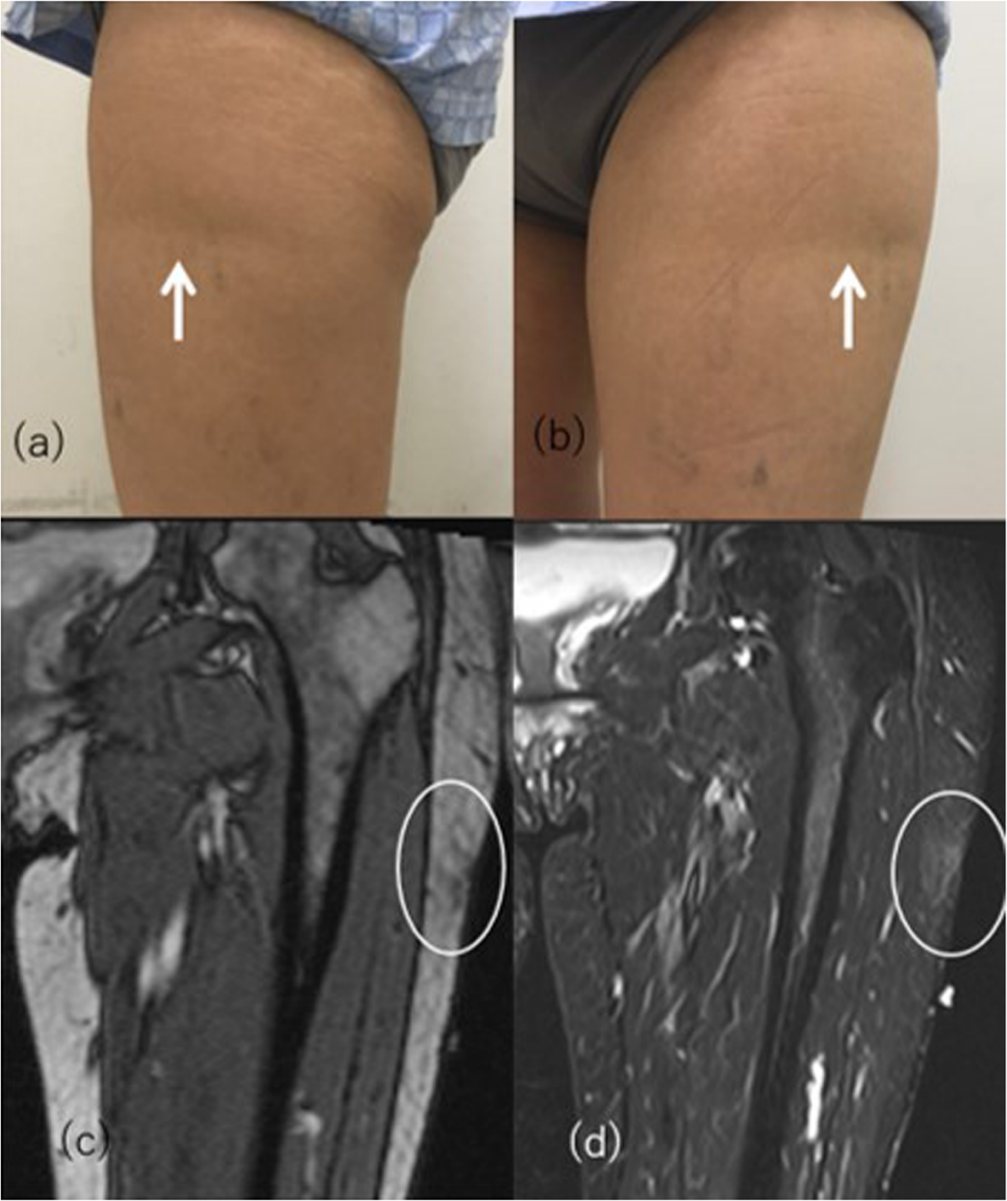
A 47-year-old woman with semicircular lipoatrophy. **a**, **b** Linear horizontal depression on the lateral aspect of left thigh at 72 cm above the ground (arrows). She had a history of working leaning against the edge of her desk daily. **c**, **d** Coronal localizer image and STIR show loss of localized subcutaneous fat tissue with slight edematous change (circle)

Semicircular lipoatrophy is a type of localized lipoatrophy characterized by band-like horizontal depressions measuring 2 to 4 cm in width, located on the anterolateral aspect of the thighs (Fig. 15). The most widely accepted cause is repetitive microtrauma against such as the edge of furniture or tight-fitting clothes [57]. Semicircular lipoatrophy is frequently reported among office working women aged 20 to 40 years. The lesion is typically observed at 72–73 cm above the ground, which is the standard height of office furniture [58]. Other risk factors are reported to include overweight, routine electrical shock, clothing made of fibers, wearing of rubber-soled shoes, and low humidity air conditioning [58]. When the suspected cause is found and removed from the daily life, the lesions resolve within 9 months to 4 years [59]. MRI shows a clear superficial loss of subcutaneous tissue without findings of panniculitis and thickening of interlobular septa with a reduction in the size of fat lobules [60].

### Others

#### Familial partial lipodystrophies

Familial partial lipodystrophy is inherited in an autosomal dominant or recessive fashion showing a mixture of partial fat atrophy and accumulation with onset during childhood or puberty [12]. The phenotypic heterogeneity of familial partial lipodystrophy has been reported as types 1–6.

#### Mandibuloacral dysplasia

Mandibuloacral dysplasia is an extremely rare autosomal recessive progeroid syndrome due to mutations in genes encoding nuclear lamina proteins, lamins A/C (LMNA) or prelamin A processing enzyme, and zinc metalloproteinase (ZMPSTE24) which has been reported in approximately 40 case reports [61, 62]. Lipoatrophy appears in childhood or early adolescence. In addition to lipodystrophy, this condition shows craniofacial and skeletal abnormalities including mandibular and clavicular hypoplasia, delayed closure of the cranial sutures, acro-osteolysis, joint contractures or bird-like facies with postnatal growth retardation, and cutaneous changes [12].

#### Acquired partial lipodystrophy (Barraquer-Simons syndrome)

Approximately 250 cases of acquired partial lipodystrophy have been reported. It typically shows a childhood or adolescent onset with a unique, cephalocaudal progression of fat loss with fat accumulation in the lower half of the body [63]. Infections, autoimmune diseases, and membranoproliferative glomerulonephritis have been linked to the development of acquired partial lipodystrophy [12].

## Adipose tumors as differential diagnoses

Adipocytic tumors are the most common soft tissue tumors clinically, and radiologists always need to differentiate them from those conditions showing localized abnormal fat distribution discussed above. Here, we illustrate imaging findings of benign and intermediate adipose tumors (lipoma, angiolipoma, spindle cell/pleomorphic lipoma, lipomatosis of nerve, and well-differentiated liposarcoma/atypical lipomatous tumor) which can be major differential diagnoses.

**Fig. 16:**
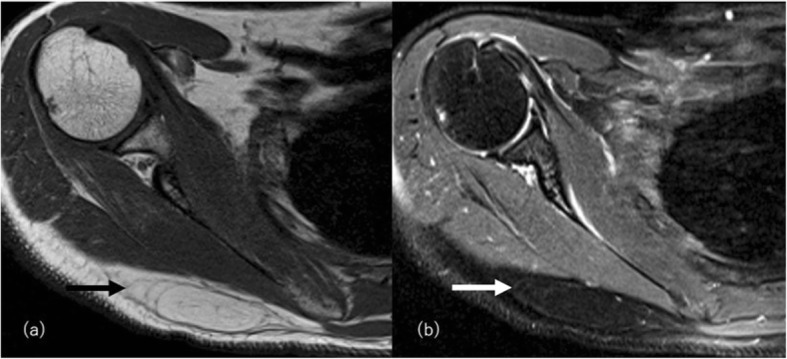
Superficial lipoma in a 60-year-old woman on the right shoulder. **a** T1-weighted image and (**b**) fat-suppressed T2-weighted show a homogeneous fatty mass (arrows) with a similar signal intensity to that of the adjacent subcutaneous fat but with a thin capsule and thin internal septa

Lipoma accounts for approximately 50% of all soft tissue tumors and the most common subcutaneous neoplasm consists mainly of mature adipose tissue [64]. Lipoma usually demonstrates an encapsulated high signal mass on T2- and T1-weighted images that contain thin septa less than 2 mm, with suppression on fat saturated images (Fig. 16), but some show a non-capsulated pattern. Lipoma commonly manifests as a solitary mass but can also manifest as multiple masses in 5–15% of cases [64]. Those atypical lipomas can resemble non-neoplastic abnormal fat distribution conditions. Lipomas are usually asymptomatic, though local pain, tenderness, or nerve compression is reported when they become large [65]. Abnormal non-neoplastic fat distribution conditions usually show no such symptoms though mechanical obstruction or metabolic dysfunction may also be present. Fat necrosis within a lipoma presents a variable imaging appearance with inflammatory or fibrotic change, and these can be difficult to differentiate from fat accumulation due to mechanical pressure or trauma, other benign adipose tumors, or well-differentiated liposarcoma.

**Fig. 17:**
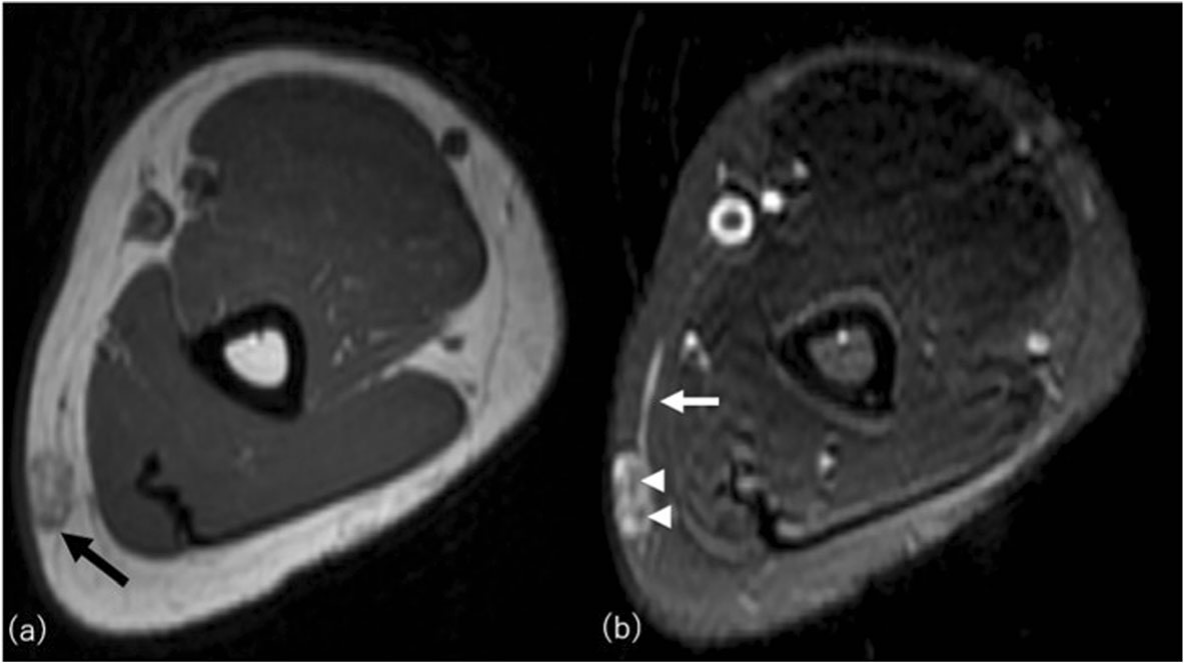
Angiolipoma in a 29-year-old man on the left upper arm. **a** T1-weighted image shows a subcutaneous tiny mass with inhomogeneous high to intermediate signal intensity (arrow). **b** Fat-saturated T2-weighted image shows hyperintense signal with focal fat suppression (arrowhead) in the mass with connection to dilated subcutaneous vein (small arrow)

Angiolipoma is a benign neoplasm consisted of mature adipose tissue and vascular structures. It represents as well-defined multiple, small subcutaneous mass with tenderness located commonly in the forearm, upper arm, or trunk [66]. The MR imaging features of these lesions are the presence of fat nodules with or without low signal on T1- or T2-weighted images with or without high signal on fat saturated T2-weighted images representing the prominent vasculature [67] (Fig. 17). Lipogranulomas found with post-traumatic lipoatrophy/lipohypertrophy would be the differential diagnosis, though the presence of pain, absence of traumatic history, and normal distribution of surrounding subcutaneous fat demonstrate the different nature of those conditions.

**Fig. 18:**
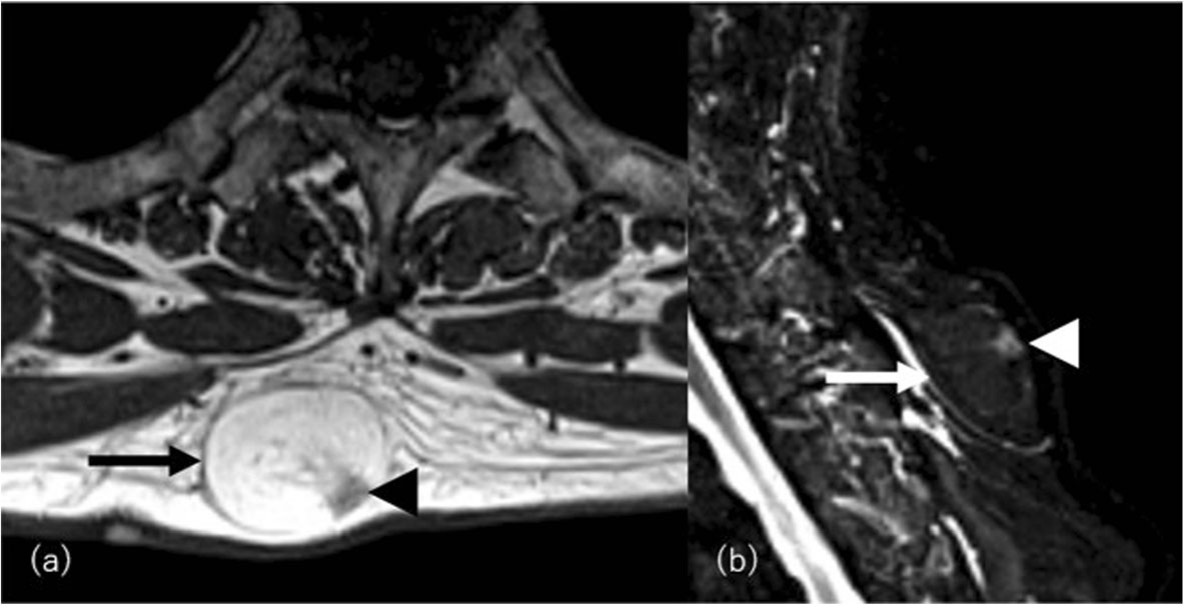
Spindle cell lipoma in a 68-year-old man with a painless mass on the posterior neck. **a** Axial T1-weighted image and **b** sagittal STIR image show a subcutaneous, encapsulated fatty mass (arrows) with amorphous non-fatty signal area (arrowheads) representing intermingled components such as collagen fibers, myxoid matrix, and vascular elements

Spindle cell/pleomorphic lipoma is a benign, well-circumscribed subcutaneous mass commonly located in the posterior neck or shoulder in middle-aged men with frequent expression of androgen receptor [68]. Histologically, spindle cell/pleomorphic lipoma consists of adipocytes with varying proportions of spindle cells with rope-like collagen bundles [69]. Fat is reported to be detectable in 89% of these lesions, ranging from 25% to 75% of the tumor volume on MRI [70]. Non-adipose components are similar to those of skeletal muscle on T1-weighted imaging and hyperintense on fat-saturated fluid-sensitive sequences (Fig. 18) Intense enhancement of the non-adipose component further supports this diagnosis. The mass is generally firmer than lipomas or most conditions with non-neoplastic localized abnormal fat distribution. When it occurs in atypical locations, it becomes challenging to differentiate from liposarcomas or even other non-adipose soft tissue tumors.

**Fig. 19:**
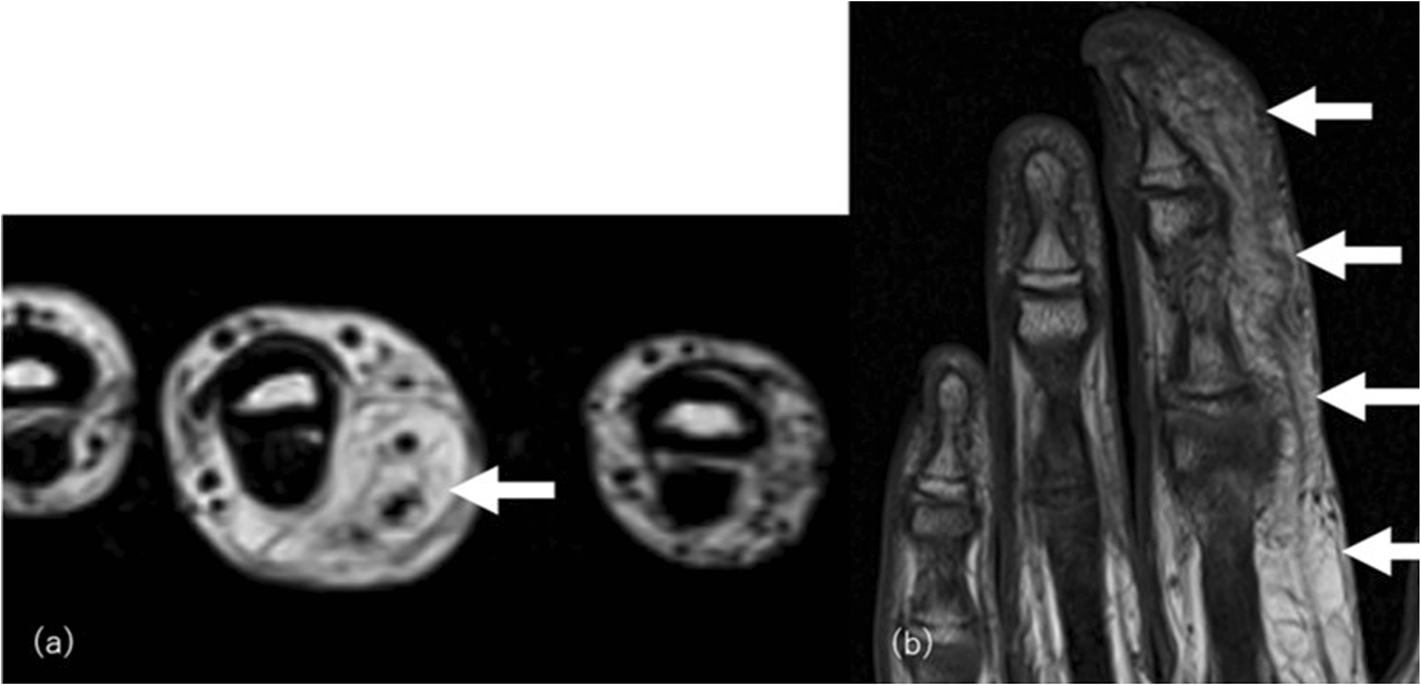
Lipomatosis of the median nerve in an 11-year-old girl without symptoms. **a** Axial T2- and (**b**) coronal T1-weighted images reveal soft-tissue hypertrophy with predominance of fat in the radial side of the middle finger along with the neurovascular structure (arrows)

Lipomatosis of a nerve is a rare, not fully understood benign condition, which has been referred to variously as fibrolipomatous hamartoma, perineural lipoma, fatty infiltration of the nerve, or neural fibrolipoma [64]. This condition most commonly occurs in patients before 30 years of age, involving the median nerve of the wrist and hand. Accompanying varying degrees of mesenchymal overgrowth including adipose tissue with frequent sensory symptoms such as paresthesia or numbness, with or without macrodactyly, is the typical presentation [71] (Fig. 19). The location and adipose tissue distribution interspersing nerve bundles are imaging findings distinctive from those of already discussed non-neoplastic abnormal fat distribution conditions.

**Fig. 20:**
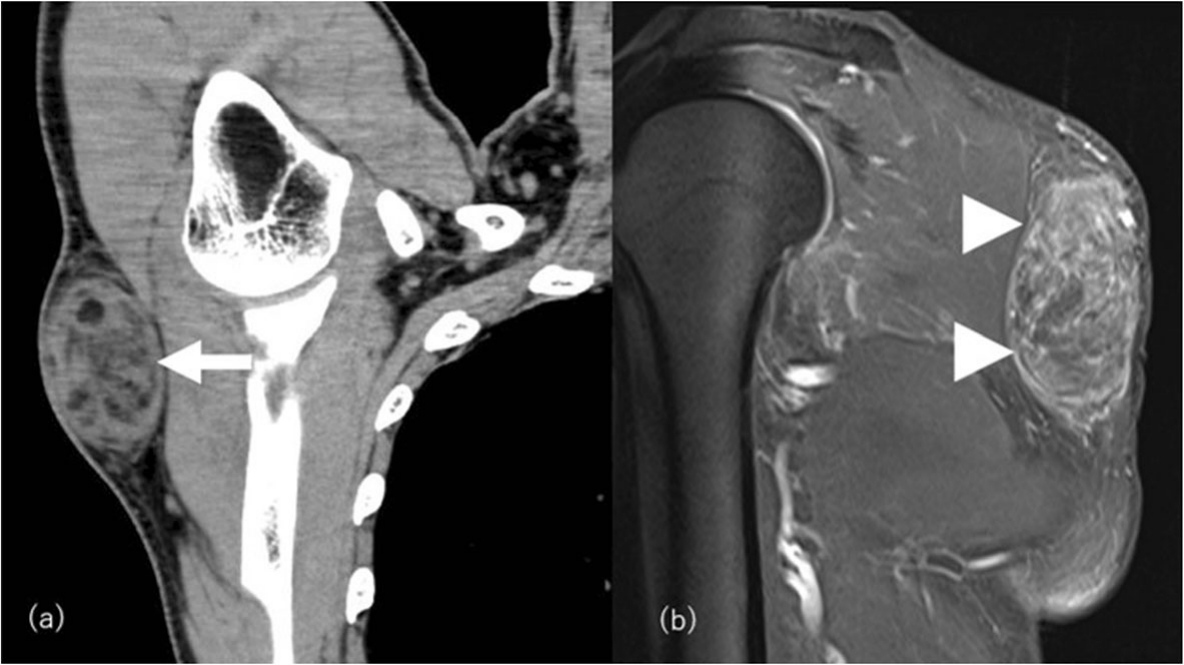
Atypical lipomatous tumor in a 62-year-old male with a painless, firm, and mobile mass gradually increasing in the right back. **a** Coronal CT image shows a fat-containing inhomogeneous density mass (arrow). **b** On post-contrast fat-saturated T1-weighted image, the non-fatty lesion shows moderate enhancement (arrowheads). Surgically diagnosed as ALT with abundant fat necrosis

Well-differentiated liposarcoma/atypical lipomatous tumor (ALT) is the most common liposarcoma, classified as an intermediate (locally aggressive) adipocytic tumor. It most commonly locates in a deep-seated well-vascularized area and rarely in subcutaneous locations [72]. Well-differentiated liposarcoma and ALT are synonyms describing similar lesions morphologically and karyotypically and are differentiated by the location of the tumor and the surgical resectability [66]. The term of well-differentiated liposarcoma is used for lesions exclusively in the retroperitoneum, mediastinum, and spermatic cord, while ALT is used for lesions arising elsewhere [66]. Well-differentiated liposarcoma/ALT can be the differential diagnosis for multiple symmetric lipomatosis, hypercortisolism, or post-traumatic lipohypertrophy with fat necrosis. The imaging findings typically depict a fatty mass with thick and irregular septa, septal enhancement, and non-adipose areas with prominent mass effect, local encasement of vital organs, and asymmetrical distribution compared with those non-neoplastic conditions [73] (Fig. 20).

## Conclusion

Radiologists should be aware of the typical imaging findings and disease spectrum of abnormal deposition of subcutaneous fat. Although the underlying conditions are diverse, the radiological findings can be the key making possible an early assessment and suggesting the optimal methods needed to achieve a definitive diagnosis.

## Data Availability

The datasets used and/or analyzed during the current study are available from the corresponding author on reasonable request.

## Abbreviations

ACTH: Adrenocorticotropic hormone
AGL: Acquired generalized lipodystrophy
ALT: Atypical lipomatous tumor
BAT: Brown adipose tissue
CGL: Congenital generalized lipodystrophy
HAART: Highly active antiretroviral therapy
HALS: HIV-1/highly active antiretroviral therapy-associated lipodystrophy syndrome
HIV: Human immunodeficiency virus
LE: Lupus erythematosus
LEP: Lupus erythematosus profundus
LScs: Localized scleroderma en coup de sabre
MSL: Multiple symmetric lipomatosis
NRTIs: Thymidine analog nucleoside reverse transcriptase inhibitors
PRS: Parry-Romberg syndrome
SCAT: Subcutaneous fat tissue
VSA: Visceral fat tissue
WAT: White adipose tissue
